# P-glycoprotein and cancer: what do we currently know?

**DOI:** 10.1016/j.heliyon.2022.e11171

**Published:** 2022-10-22

**Authors:** Carlos Pilotto Heming, Wanjiru Muriithi, Lucy Wanjiku Macharia, Paulo Niemeyer Filho, Vivaldo Moura-Neto, Veronica Aran

**Affiliations:** Instituto Estadual do Cérebro Paulo Niemeyer (IECPN), R. do Rezende, 156 – Centro, Rio de Janeiro, 20231-092, Brazil

**Keywords:** MDR phenotype, P-glycoprotein, ABCB1, Cancer biomarkers, ABC transporters

## Abstract

Acquired resistance during cancer treatment is unfortunately a frequent event. There are several reasons for this, including the ability of the ATP-binding cassette transporters (ABC transporters), which are integral membrane proteins, to export chemotherapeutic molecules from the interior of the tumor cells. One important member of this family is the protein known as Permeability Glycoprotein (P-Glycoprotein, P-gp or ABCB1). Its clinical relevance relies mainly on the fact that the inhibition of P-gp and other ABC transporters could result in the reversal of the multidrug resistance (MDR) phenotype in some patients. Recently, other roles apart from being a key player in MDR, have emerged for P-gp. Therefore, this review discusses the relationship between P-gp and MDR, in addition to the possible role of this protein as a biomarker in cancer.

## Graphical abstract

**Figure:**
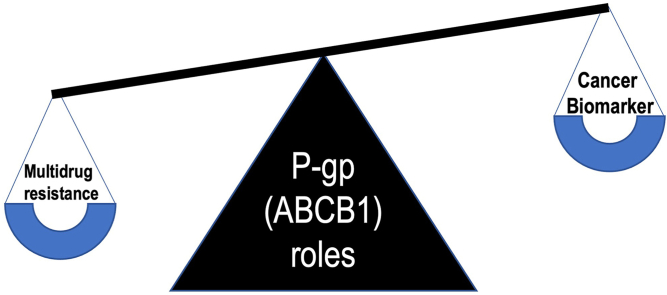

## 1 Introduction

The success of cancer therapy depends vastly on the control of inherent or acquired resistance of tumor cells to chemotherapeutic agents. Resistance to one drug often can lead to drug resistance events due to a phenomenon known as multidrug resistance (MDR), which can be described as the ability of cells to either be intrinsically resistant or acquire resistance to several structurally unrelated molecules that do not share a common mechanism of action. This multifactorial phenotype relates to inefficient cancer treatment and poor patient prognosis [1, 2].

Some cancers, such as gastrointestinal and renal cancers are unresponsive to chemotherapy having a high degree of intrinsic MDR. Others, such as leukaemia and lymphoma, often respond to initial treatment, eventually gaining resistance that facilitates tumor progression [3].

The cellular mechanisms behind multidrug resistance are varied and, most times, not well understood, as they may arise through genetic or epigenetic changes that alter drug delivery and sensitivity [4]. These mechanisms range from mechanical barriers to biochemical aspects, such as but not limited to intracellular inactivation of cytotoxic drugs [3], the existence of quiescent cells with stem-like characteristics that evade anticancer drug action [1], and overexpression of efflux pumps [5]. The role of pumping out drug molecules, thus decreasing their concentration in the cells, notably revolves around the adenosine triphosphate-dependent family of transporter proteins ATP-binding cassettes (ABC transporters) [5]. By actively removing molecules from the interior of the neoplastic cells, efficiency of chemotherapy is reduced.

ABC transporters are present in all kingdoms of life. Although they function both as ATP-dependent importers and exporters in bacteria, eukaryotic ABC proteins function solely as efflux pumps [6]. In humans, 48 genes and 1 pseudogene that encode ABC transporters have been previously identified and are grouped into seven families that range from ABCA to ABCG depending on their homology and domain organization, 12 of which are related to drug efflux and subsequent MDR [7]. ABC transporters are also involved in the physiological efflux of lipids, sterols, peptides and toxins. P-glycoprotein (P-gp) is the most studied and well-characterized ABC transporter associated with resistance to cancer chemotherapy [8]. In this review we sought to discuss the relationship between P-gp and cancer, beyond simply MDR events.

## 2 P-glycoprotein expression and role in multidrug resistance mechanisms

The first ABC transporter described was the Permeability Glycoprotein (P-Glycoprotein or P-gp) or ABCB1, in 1976 by Juliano and Ling. By labeling cell-surface carbohydrates, they identified a surface phosphoglycoprotein expressed in colchicine-resistant Chinese hamster ovary cells [9]. It is a 170 kDa transmembrane glycoprotein, which prevents certain substance's accumulation in the intracellular environment by actively stimulating the output of these substances [10].

P-gp is predominantly found in the apical membranes of epithelial cells throughout the body and is endogenous to the gastrointestinal tract, liver, kidneys, testicles, ovaries, adrenal and pituitary glands, placenta, choroid plexus, and the capillaries of the brain (Table 1). It is present in the blood luminal membrane of the brain capillary endothelial cells that make up the blood–brain barrier (BBB), where it serves a protective role limiting the uptake of xenobiotics [11, 12, 13, 14, 15]. P-gp is also expressed on CD34 + hematopoietic progenitor cells, natural killer (NK) cells and CD8+ T cells [16]. P-gp is frequently found in cancer stem-like cells (CSCs). Table 1 summarises the main tissues in which P-gp was found to be expressed.

**Table 1:** Tissues in which P-gp is expressed and has physiological efflux roles.

Tissue	P-gp expression sites	References
Adrenal gland	Glandular cells in medulla and cortex	[17]
Basal ganglia	Glial cells	[18]
Cerebral cortex	Blood luminal membrane of the brain capillary endothelial cells	[11]
	End-feet of astrocytes	
Colon	Apical surface of columnar epithelial cells	[17]
Fallopian tubes	Apical portion of epithelial cells	[19]
Gallbladder	Glandular cells	[18]
Hippocampus	Microglia	[20]
Kidney	Renal proximal tubular cells	[21, 22]
Liver	Cholangiocytes	[17, 18]
	Biliary canalicular surface of hepatocytes	
Ovaries	Follicle cells, oocytes, somatic cells of the ovary	[19, 23]
Pancreas	Apical surface of small ductules	[17]
Placenta	Apical microvilli membrane of syncytiotrophoblast	[21, 24]
Small intestine	Glandular cells	[17, 21]
	Apical membranes of epithelial cells	
Testicles	Endothelial capillary cells	[22]
	Leydig cells	
	Early spermatids	

**Figure 1:**
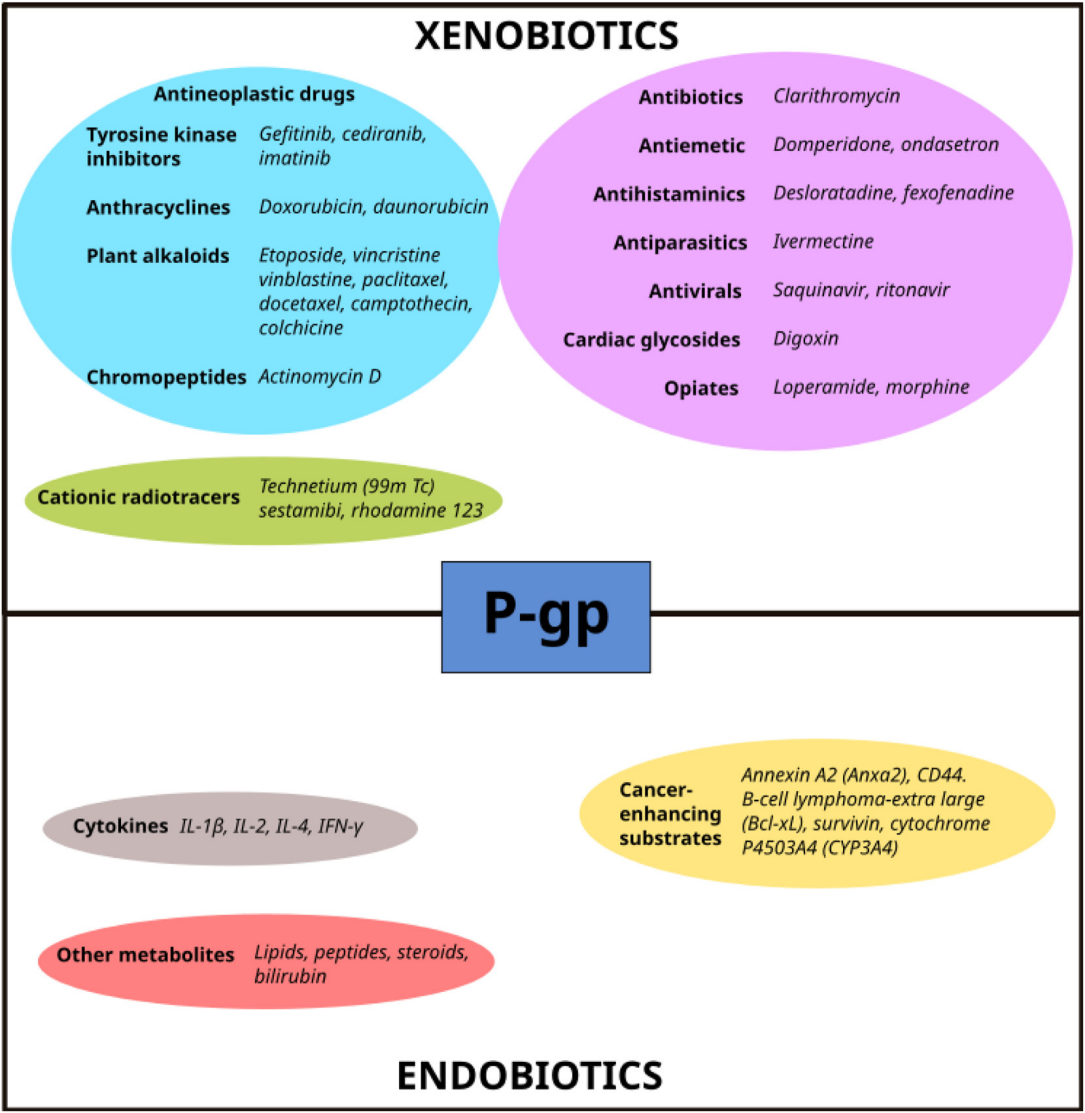
The diverse array of substrates transported by P-gp can be divided into xenobiotics and endogenous compounds, which can be further classified into distinct groups as shown.

P-gp actively transports large hydrophobic and amphipathic, positively charged molecules. It extrudes a variety of lipophilic drugs, most notably antineoplastic drugs such as the *Vinca* alkaloids vinblastine and vincristine, the anthracyclines doxorubicin and daunorubicin, the epipodophyllotoxin etoposide, camptothecin and taxanes paclitaxel and docetaxel [25, 26, 27]. Besides cytotoxic drugs, P-gp also transports several other exogenous compounds including opiates, polycyclic aromatic hydrocarbons, technetium (99mTc) sestamibi, rhodamine 123 [2,28], cardiac drugs (digoxin), antiparasitic molecules (ivermectin), antiemetic (domperidone and ondansetron) [29] and antihistamine medications (desloratadine and fexofenadine) [30, 31]. Among endogenous compounds carried by the protein, certain cytokines, corticosteroids (aldosterone and hydrocortisone), lipids, bilirubin, bile acids and platelet activating factors can be pointed out [32]. Different cancer-enhancing substrates are also P-gp substrates, as reported in Figure 1. The transporter has been linked to other roles besides MDR, such as the inhibition of apoptosis [33]. Figure 1 shows examples of xenobiotics and endobiotics transported by P-gp.

## 3 P-gp structure

**Figure 2:**
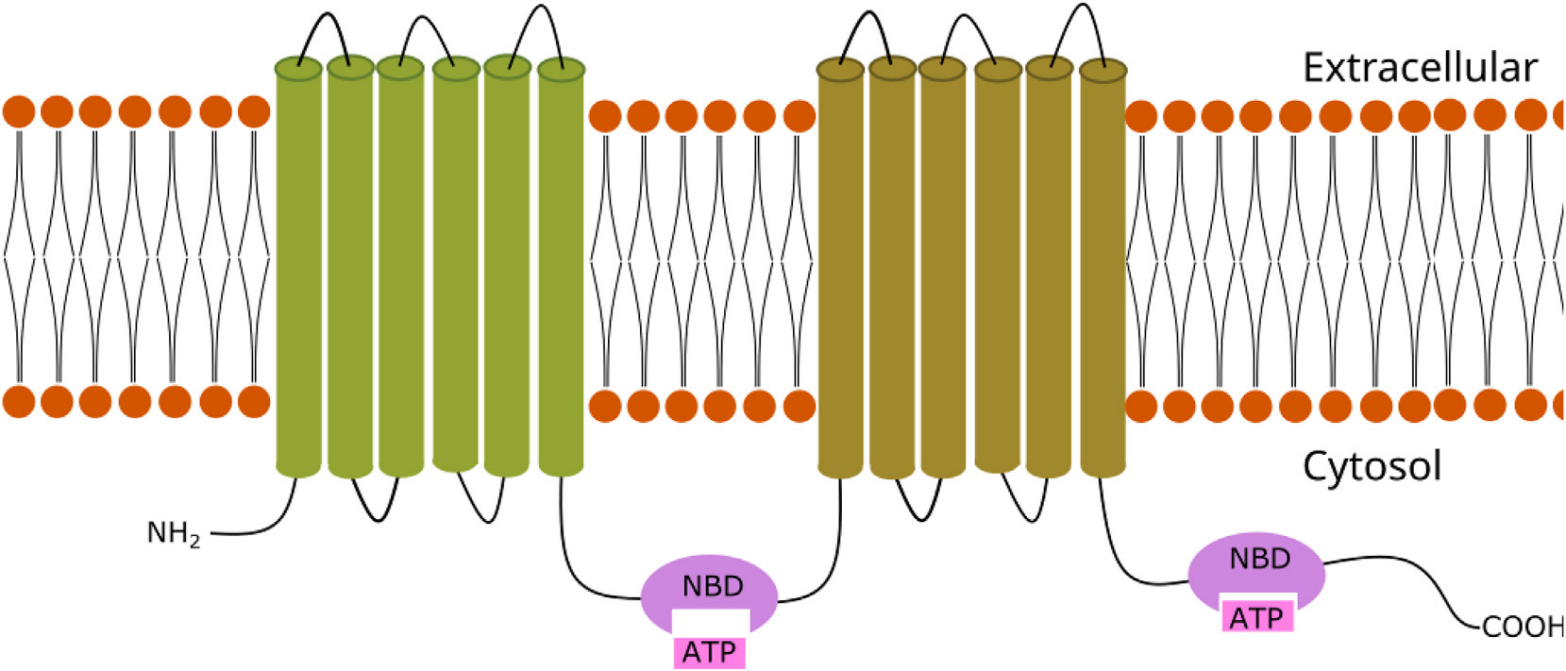
Example of P-glycoprotein's transmembrane domains (TMDs) structure, showing two nucleotide binding domains (NBD) binding to ATP molecules. The membrane spanning cylindrical objects represent the alpha helices protein domains.

Structurally, P-gp consists of four domains, all common to the ABC transporter superfamily. It includes two cytoplasmic nucleotide-binding domains (NBDs), with one ATPase site each, and two transmembrane domains (TMDs), each containing six transmembrane alpha-helices that recognize and bind the substrate molecules [34, 35], as exposed in Figure 2. The two homologous halves of the protein are connected by a central sequence known as the linker region [36].

Currently, the most widely accepted model to explain the mechanics of substance transport is the “alternating access” one [37]. P-gp undergoes large conformational changes during the efflux cycle. The substrates interact with the transporter in an inwards-facing conformation, in which there is a large degree of separation between the NBDs and the translocation pathway is reachable from the cytoplasm [34]. Substrate binding then signals the free NBDs to bind to two ATP molecules, which leads to its dimerization and subsequent switch in the transporter's topology, which adopts an outwards-facing conformation, thus extruding the molecules [38, 39].

The molecular structures of P-gp have been more recently investigated using X-ray crystallography. This has evidenced the existence of flexible inwards-facing conformations, depending on the stimuli. For example, larger substrates may induce an enlargement of the TMD-built portal-like structures [37].

Given its clinical importance, it was hypothesized that the inhibition of P-gp and other ABC transporters could result in the reversal of the MDR phenotype in patients. However, the results observed *in vitro* could not be replicated clinically, as three generations of inhibitors that are pharmacologically active have been developed, but all leading to varying degrees of toxicity. To date, no inhibitor has been shown to safely and completely reverse MDR in human clinical trials, especially due to issues with pharmacokinetic or pharmacodynamic interactions and toxicities [39, 40].

## 4 P-glycoprotein expression in tumors

The development of certain tumors appears to be related to the expression of P-gp, even in tissues in which it is normally absent (e.g., neuroblastoma and chronic myeloid leukaemia) [41]. The upregulation in cancer cells was described as an adaptive response to evade chemotherapy-mediated cell death [12]. However, due to the transporter's ability to transport a wide variety of substrates, enhanced expression in CSCs and an increased mitochondrial ATP output, it has been proposed that in addition to exporting drugs from cells, ABC proteins also transport cell-signaling molecules that contribute to tumorigenesis [42]. For example, the ABCB1 gene is induced not only by anticancer agents, but also by many other exogenous stimuli, such as heat shock stress and UV irradiation [43, 44]. While most solid tumors induce expression of P-gp as a response to treatment, cancers might have enhanced genetic and epigenetic modulators that lead to increased constitutive expression of the transporter [35].

P-gp is present in cancers derived from epithelial tissues that physiologically express the transporter such as liver, colon, and kidney [12]. Interestingly, lower than normal P-gp levels were shown to be an early event in the colorectal adenoma carcinoma sequence, suggesting promoting carcinogenesis through increasing intracellular exposure to P-gp substrates [45].

Tumor types in which P-gp levels were low at diagnosis, such as leukaemia, lymphoma, and multiple myeloma, often overexpress the transporter after chemotherapy and cancer recurrence [12]. In the case of acute myeloid leukaemia, P-gp is expressed in approximately 30% of patients at diagnosis and in most cases at relapse [46].

Studies have also demonstrated that intrinsic P-gp expression levels in non-small cell lung cancer (NSCLC) are similar to normal epithelia, although there is significant activation of its expression during chemotherapy [47], as well as in bladder cancer [48]. Positive regulation of P-gp expression in these cases is often correlated with shorter survival rates [49].

Although counterintuitive due to its prominent role in cancer resistance, P-gp has been linked to favorable prognosis. One example is prostate carcinogenesis, shown to induce histone modifications and DNA methylation in the ABCB1 gene, resulting in its silencing [50, 51]. Despite this, ABCB1 gene methylation has been correlated with P-gp upregulation in renal cell carcinoma [52]. Another evidence that seems contradictory at first is the evidence that high P-gp expression plays a role in protection of the normal bladder urothelium from carcinogen exposure, thus preventing tumorigenesis [53]. These findings indicate high expression of P-gp is more frequent in tissues of the normal bladder and high-grade carcinoma with the lowest expression levels present in low-grade bladder carcinoma.

In the brain, capillary endothelial cells formed by brain tumors express P-gp in 80% of patients, and the tumor cells itself express P-gp in 20% of patients. Interestingly, it is not found in the neovasculature in other primary tumors [54]. Low-grade gliomas such as pilocytic astrocytoma and oligodendroglioma, all demonstrate P-gp vascular staining. Vascular endothelial staining is also present in anaplastic gliomas (anaplastic astrocytoma, oligodendroglioma and ependymoma) [55].

In glioblastoma cells, P-gp is expressed heterogeneously at both membrane and cytoplasm. In secondary glioblastoma, P-gp expression appears in focal groups of tumor cells [55]. Interestingly, P-gp is not commonly found in metastatic brain tumors [55, 56]. Primary neuroblastomas originating from the adrenal glands show a heterogeneous vascular staining pattern like that seen in the high-grade primary brain tumors [55]. Table 2 summarizes the P-gp expression status according to distinct tumor types, as suggested by different published studies.

**Table 2:** P-gp expression status according to tumor type.

P-gp expression status	Tumor type	References
Upregulation	Acute myeloid leukaemia	[57, 58]
Upregulation	Adrenocortical carcinoma	[59]
Upregulation	B-cell lymphoma	[60]
Upregulation	Bladder carcinoma	[53]
Down-regulation	Breast cancer	[61, 62]
Upregulation	Cervical carcinoma	[63, 64]
Upregulation	Chronic myeloid leukaemia	[41]
Down-regulation	Colorectal adenocarcinoma	[25, 65]
Upregulation	Gallbladder carcinoma	[66]
Upregulation	Glioma	[67, 68]
Upregulation	Hepatocellular carcinoma	[69, 70]
Upregulation	Neuroblastoma	[41]
Upregulation	Ovarian cancer	[71]
Upregulation	Plasma cell myeloma	[72]
Down-regulation	Prostate cancer	[51, 73]
Upregulation	Renal cancer	[74]
Down-regulation	Schwannoma	[55]
Upregulation	Small cell lung cancer	[75]

## 5 Relevance of P-glycoprotein in brain tumors

There are not many publications regarding the expression of P-gp in brain tumor tissues. P-gp has the highest expression in brain capillary endothelial cells among all known multidrug resistance-related proteins [76]. Its presence not only results in resistance to chemotherapy agents in patients with cancer of the Central Nervous System (CNS), but it also contributes to poor penetration in the blood-brain barrier (BBB), the diffuse barrier that impedes influx of some compounds from blood to brain [77]. It can be found at both the endothelium as well at the astrocyte end-feet at the microvasculature, and in perivascular astrocytes. Expression of P-gp in brain tumor cells is weak compared to vessels [76, 78], being mostly present in glioblastoma and meningeal sarcoma cells. Interestingly, they are often absent in meningeal sarcoma vasculature [67].

Another clinically relevant ABC transporter is the multidrug resistance-associated protein 1 (MRP-1) or ABCC1, a glutathione S-conjugate efflux pump that is also expressed on plasma membranes [79]. In contrast to P-gp, in the context of brain tumor, MRP-1 expression is restricted to glial tumor cells and endothelial expression has not been detected in neither normal nor neoplastic vasculature [67, 68]. While expression of P-gp is associated with the BBB, MRP-1 serves a different protecting function in the brain. It is believed to contribute to the blood-cerebrospinal fluid barrier, as it has been detected in the epithelium of the choroid plexus and in ependymal cells of the ventricles [80].

P-gp is differentially expressed among different CNS tumors. In glioblastoma, for example, P-gp is present in 90% of primary and 60% of secondary [68]. In this tumor type, the modulation of P-gp expression following chemotherapy to date is controversial. Indeed, Tews *et al.* noted constant expression before and after adjuvant treatment, suggesting it is predominantly intrinsic in glioblastoma [68]. However, Abe *et al.* have reported an increase of P-gp expression ratio after glioblastoma chemotherapy [67]. Following immuno-staining in glioblastoma, P-gp appears in a diffuse, fibrillary expression pattern in the white matter of the brain with a strong presence in the neovasculature of the tumor [68]. P-gp expressing CSCs have been reported in the perivascular spaces of glioblastoma patients [10].

Expression in lower-grade gliomas, such as WHO grades II and III, correspond to 45% and 55% respectively [68]. This indicates a possible correlation between P-gp expression levels and glioma aggressiveness [81], with fine granular expression in the cytoplasm [68].

Demeule *et al.* detected P-gp by Western blot analysis in 60 human brain tumors, including meningiomas, schwannomas and both low- and high-grade gliomas [56]. Most remarkable were in schwannomas whose expression levels were reduced by 65% when compared to normal brain tissue expression, and meningiomas, in which levels were found to be over 10-fold higher [56]. In oligo-astrocytomas, the multidrug resistance-related factor is expressed in both oligodendroglial and astrocytic tumor cells [55]. Brain metastases from melanomas and lung adenocarcinomas had lower P-gp levels (70% and 95% lower, respectively), when compared to normal brain tissue [56].

Regarding other CNS regions, P-gp has been also identified in the human pituitary gland, localized in the capillaries, pituicytes, and anterior pituitary cells, and in prolactin- and growth hormone–releasing pituitary tumor cell lines [15]. It has also been detected in HCG-producing cystic craniopharyngiomas [82]. Primary neuroblastomas originating from the adrenal glands showed a heterogeneous vascular staining pattern like that seen in the high-grade primary brain tumors [55].

Temozolomide (TMZ), which is the drug of choice in current high-grade glioma treatment, is generally believed to infiltrate the BBB relatively well. In fact, penetration in the brain is sufficiently high to improve the median survival of glioblastoma patients [83]. However, studies such as by the van Tellingen group demonstrated that P-gp and other ABC transporters are limitants to its total penetration [84]. As a matter of fact, other recent studies have indicated TMZ as a possible target for P-gp-mediated efflux [85]. In addition, temozolomide treatment seems to upregulate P-gp expression by inducing the production of Epidermal Growth Factor (EGF) [86], process which will be discussed further into the review.

Seizure control is often addressed in patients with low-grade glioma with the use of antiepileptic drugs [87]. Generally, these pharmaceuticals must traverse the BBB and act as substrates for P-gp [88]. Therefore, P-gp imposes a challenge to both neoplasm regression and symptom control. In the last decade, some studies have inclined to a possibility of addressing TMZ as initial treatment in cases of uncontrolled epilepsy [87], having its success perhaps due to saturation of the efflux pumps.

## 6 P-glycoprotein mechanisms of resistance against cancer treatments

A large variety of molecules with vastly different chemical structures and molecular weights are known to efflux through the P-gp transporter. Studies using murine models have shown that the drug-binding site in the inwards-facing orientation of P-gp is made up of both hydrophobic residues and few polar side chains, which explains the diverse array of substrate binding [35, 89]. Recently, Lee and co-workers have identified 55 novel substrates of P-gp, adding to a total of 90 known compounds that bind to the transporter [90].

Drug resistance is a multifactorial phenomenon that results in therapeutic failure in a variety of cancers. Growing evidence suggests that oral chemotherapy is preferable to intravenous administration due to its lower cost, prolonged antitumor activity, reduced toxicity and increased patient compliance. However, presence of P-glycoprotein along the gastrointestinal tract (GIT) including the small intestine or the primary site for the epithelial absorption has been shown to reduce their oral bioavailability [91].

Despite its presence in the gut, drug efflux transporters seem much more potent in restricting the entry of substrate drugs into the brain than preventing uptake from the GIT. Substrate drugs with an excellent oral bioavailability (>90%) such as imatinib, which is used in therapy for Philadelphia chromosome-positive chronic myelogenous leukaemia and gastrointestinal stromal tumors have poor brain penetration that have been shown to significantly enhance in ABCB1−ve mice [88, 92, 93].

P-gp also modulates expression of cytochrome P4503A4 (CYP3A4) that may in turn deactivate some anticancer drugs [94]. It is also involved in subcellular resistance modulation, as P-gp has been shown to be localized in the Golgi apparatus and in the rough endoplasmic reticulum in drug-resistant cells. In equivalent proportions, it is expressed in the mitochondria cristae, where it is involved in drug accumulation in the organelle but not in its efflux. This suggests an orientation in the mitochondrial membrane inverse to the plasma membrane, thus protecting the nucleus from cytotoxic activity [95]. Further preventing nuclear degradation, the protein is also distributed along the nuclear envelope [96]. More studies should be performed to investigate the role of P-gp in cancer treatment.

## 7 P-glycoprotein interactions that promote MDR and aggressive tumor phenotypes

The interaction of P-gp with several other membrane moieties has been studied in an effort to unravel the different, often contradictory function of the transporter in cancer. Recent body of works, including our own [38] have sought to identify the roles of ABC transporters outside drug efflux mechanisms that lead to poor diagnosis in patients. Many studies have observed that drug-resistant cancer cells always displayed a more invasive phenotype compared to parental tumor cells, however, the link between cells overexpressing ABC transporters after drug exposure and a more aggressive tumor phenotype is still not clear. However, activation of P-gp promoter associated with its overexpression in tumors has been shown to correlate with increased lymph node metastasis in breast cancer [97].

One of the more poignant interactions of P-glycoprotein is with the CD44 protein, an adhesion molecule of the hyaluronan (HA) receptor family of cell-surface glycoproteins implicated in cell motility, adhesion, and metastases [98]. A study by the Rodriguez group showed that there was a correlation between the expression of P-gp and CD44 and further, that one protein directly influences the expression of the other. The two proteins were found to co-localize within the cell membrane and the disruption of this interaction was shown to markedly reduce drug resistance, cell migration, and in vitro invasion [99]. In addition, the transgenic expression of CD44 in cells increases P-gp expression and knockdown of CD44 interferes with the drug efflux mechanism of P-gp-mediated MDR [100].

Another protein, Annexin A2 (Anxa2), which has been found to co-localize and co-immunoprecipitate with P-gp in MCF-7/ADR cells, interacts with P-gp resulting in enhanced malignant phenotype of cancer cells [101, 102]. The upregulation of this calcium-dependent phospholipid-binding protein has been shown to correlate with increased cell proliferation, cell motility, actin rearrangements, angiogenesis and metastasis in different cancers [103]. In addition, it is significantly associated with rapid recurrence after chemotherapy [104]. A study in breast cancer showed increased cell motility after drug exposure correlated with upregulation of Anxa2 phosphorylation in a dose and time dependent manner [105]. This effect was inhibited by P-gp knockdown using small interference RNA or by administration of P-gp inhibitors. This study unraveled a possible role for P-gp in signal transduction possibly by interaction with a Src kinase leading to the augmentation of Anxa2 phosphorylation and increased invasiveness in cancer cells.

The association of P-gp and inhibition of cell death in cancerous cells has also been reported in several studies including in hepatocellular, colorectal, prostate cancer, and gastric cancer. The question remains whether P-gp is involved in this process through transduction of death signals or through protein-protein interactions with entities known to play a role in the death signal cascade. P-glycoprotein has been shown to interact with Bcl-xL protein to protect cells from caspase mediated death. In a study using *Helicobacter pylori*-related gastric cancer, P-gp was found to be often co-localized with Bcl-xL on the mitochondrial membrane but absent in normal gastric mucosa cells [106]. Moreover, the selective silencing of P-gp by siRNA resulted in a significant rise of the cell apoptotic index in gastric cancer cell lines exposed to oxidative stress. Although its exact mechanism in anti-apoptotic signaling is unknown, it has been shown to be caspase-dependent, with its expression resulting in decreased active caspase 3, as in a study involving acute T-cell leukaemia cell lines [107].

In addition, survivin, a member of the inhibitor of apoptosis (IAP), which blocks apoptosis induced by a variety of apoptosis triggers was shown to be regulated by P-gp at the transcriptional level [108]. The study demonstrated that verapamil, a specific inhibitor of P-gp, could abrogate survivin promoter activity in breast cancer MCF-7/ADR cells. This was postulated to be via changes enacted by P-gp activity on the PI3K-Akt pathway.

## 8 Immune cell regulation by P-gp

P-glycoprotein has been associated with cell motility in both malignant and normal immune cells. Expression of P-gp in immune cells has functional consequences on their migration, cytotoxic role, survival and differentiation. High expression has been shown in NK cells, B cell, dendritic, macrophages, CD4+ and CD8+ T cells [109]. Furthermore, it has been shown that P-gp is required for DC migration as it mediates the production of an unidentified substrate triggering a migration signaling [110].

In NK cells, treatment with verapamil, a P-gp inhibitor, decreases their cytotoxic functions in a dose-dependent manner [111]. Tumor-associated macrophages (TAMs) and microglia are known to polarize either to pro-inflammatory (M1) or anti-inflammatory (M2) phenotypes in response to environmental stimuli [112]. The M2-phenotype, which is pro-tumor, facilitates cell growth, migration and angiogenesis by producing several tumor-supporting factors [113]. A study showed that the M2 phenotype displayed higher expression of P-glycoprotein compared to the M1-phenotype [114] which may play an important role in not only drug resistance mechanisms in the tumor microenvironment (TME) [115] but also other non-drug efflux related roles discussed above. It would be interesting to investigate the role of P-gp upregulation in glioma and in particular glioblastoma, which is known to be highly infiltrated with M2-phenotype microglia to determine if the transporter has a role in the infiltration of these immune cells in the tumor or their polarization towards the pro-tumor M2 phenotype.

Evidence point to the fact that P-gp has a role in immunosurveillance, a process by which the immune system protects the organism against tumor development [116]. One example is that P-gp has been shown to be involved in the excretion of the inflammatory cytokine tumor necrosis factor-α (TNF-α) in T-cells [117]. The exact role of P-gp in excretion of cytokines remain to be elucidated, whether it is direct or indirect. Evidence is, in pharmacological inhibition of P-gp TNF-α levels are reduced, and there is enhancement of interleukin-6 (IL-6) [118]. Elevated IL-6 could indicate inflammatory response, observed during chemotherapy for example [119].

## 9 Examples of P-glycoprotein-modulating signalling pathways in cancer

Although the signaling pathways that modulate P-gp's response are still to be fully elucidated, oncogenic signaling molecules such as nuclear factor kappa B (NF-κB), Akt, phosphoinositide 3-kinase (PI3K) and cyclooxygenase 2 (COX2) together with the transporters play a major role in chemoresistance and survival, as well as CSCs renewal and differentiation [42]. One example is the regulation of P-gp by CD133 and DNA dependent protein kinase (DNA-PK) via the PI3K/Akt–NF–κB pathway in MDR glioblastoma cells [120]. Inhibition of the PI3K signaling by LY294002 in osteosarcoma was shown to inhibit P-gp and ABCC4 expression, interrupting the stem cell cycle and inducing apoptosis [42].

A study by Katayama *et al.* suggests the ubiquitin-proteasome pathway regulates the degradation of P-glycoprotein [121]. Huang *et al.* have demonstrated that P-gp expression in renal cell carcinoma stem-like side population is directly regulated by protein kinase C-epsilon (PKCε) through the PI3K/Akt and MAPK/ERK pathways [122].

Transcription factors such as c-Jun and c-Fos have been shown to also bind to its promoter to regulate transcription of P-gp. Their activation by the MAPK/ERK pathway has been correlated to P-gp upregulation in GBM following TMZ treatment. EGF secretion is induced by the treatment, resulting in Epidermal Growth Factor Receptor (EGFR) activation, which may act as an initiator of the MAPK pathway [86].

In another study involving osteosarcoma cells, Chen *et al.* demonstrated in 2019 that the inhibition of estrogen-related receptors alpha (ERRα) increased drug sensitivity via regulation of ABCB1. This has been observed as ERRα binds to the transcription factor of SP3, increasing the transcription of P-gp [123]. The aforementioned study also suggested that miR-9 is involved in ERRα-regulated mRNA stability of this transporter [123]. Another microRNA involved in P-gp regulation is miR-138 whose upregulation in vincristine-resistant leukaemia cell line HL-60/VCR may reverse the resistance by downregulating ABCB1 mRNA and subsequent P-glycoprotein, according to a study by Zhao *et al.* [124].

Pharmacological inhibition frequently targets both P-gp and ABCG2 (BCRP) at once, sensitizing breast cancer to doxorubicin, and endothelial ovarian cancer cells to paclitaxel and cisplatin [42, 125]. Similarly, bone metastasis of prostate cancer cell line PC3 treated with cyclopamine, a Smoothened (SMO) signaling inhibitor, downregulated the expression of both P-gp and BCRP [1].

## 10 Clinical trials with P-glycoprotein

Currently, there are no approved drugs available for cancer treatment that reverse MDR phenotype by specifically targeting P-gp. Despite successful attempts of MDR reversal in cell culture settings, the shortcoming in the clinical setting is associated with low specificity and high toxicity levels [126]. The toxicity can be interpreted as a result of the dosage required for P-gp inhibition that leads to unacceptable side effects due to non-selective distribution in the body upon delivery, as well as the co-administration with anticancer drugs which may be noxious [127]. Gottesman *et al.*, addressed this subject in 2002 by suggesting that normal tissues could be protected from inhibitory toxicity by drug-resistance gene transfer [128].

Recently, efforts have been concentrated on two options: the development of a new generation of P-gp inhibitors with improved selectivity, and novel delivery approaches that prevent inhibition in non-target organs. Several first and second-generation inhibitors such as verapamil, quinine, Cyclosporin A and valspodar were first observed as P-gp substrates [129, 130] and also inhibit CYP3A enzymes, meaning they have the potential to alter the pharmacokinetics of many anticancer agents, thus making the MDR reversal ineffective [131]. Later, new agents with higher specificity for P-gp were developed. These make up the third generation of P-glycoprotein inhibitors and have minimal effect on other membrane transporters and CYP3A. They are effective at lower doses and showed promise, especially in the case of tariquidar (XR9576). Its inhibitory effects were superior compared to older generations and did not interfere with doxorubicin or paclitaxel when administered in patients with solid breast tumors, as shown by Pusztai and co-workers [132], albeit with limited clinical success in restoring drug sensitivity. In addition, recent studies employing tariquidar presented toxicity and had phase III trials abandoned [133].

Other third generation inhibitors are yet to show any clinical benefit. Examples include a phase III trial with zosuquidar (or LY-335979) that did not show improved outcomes in acute myeloid leukaemia patients [134]. Success in clinical trials of P-gp inhibitors were also affected by a lack of tumor penetration in some cases [132, 135]. Other reports suggest a potential target in the nucleotide binding domains of P-gp [36], which some have stipulated could result in inhibitors becoming substrates of the transporter [126].

Tyrosine kinase inhibitors (TKIs) such as gefitinib are among studied targeted therapy alternatives in glioblastoma that have failed and identified as substrates of efflux transporters P-gp and BCRP [14, 136]. Naturally, efforts have been made to combine TKIs with P-gp inhibitors as potential therapy for glioblastoma, for instance the co-administration of cediranib with ketoconazole [137].

Directly inhibiting P-gp function has encountered major obstacles to overcome MDR in the clinic. Therefore, alternative strategies have been developed which target the expression of P-gp. Post-transcriptional gene silencing approaches may offer higher efficacy and specificity for downregulation of P-gp. These strategies include antisense oligonucleotides, ribozymes, and RNA interference, which are effective in *in vitro* assays but with limited success in *in vivo* models and are not currently in the clinical trials [127].

## 11 Conclusion

ABC transporters have been linked to MDR mechanisms for a long time. However, their exact role in other cellular events such as in tumorigenesis processes is still not well understood. Different ABC transporters have been associated with variable expression rates in tumor tissues, which could indicate a role in tumorigenesis. This review sustains that P-gp is important in this context based on previously published data on different tumor types. It also draws attention towards the potential relevance of P-gp in brain tumors, showing that there is a lack of enough data concerning P-gp in this tumor type. Interestingly, the levels of P-gp expression were found up- and down-regulated depending on the tumor tissue analyzed, suggesting that there might be a tissue-specific pattern of gene expression. Extra efforts are needed to try to uncover the precise role of MDR proteins in tumor biology.

## Declarations

### Author contribution statement

All authors listed have significantly contributed to the development and the writing of this article.

### Funding statement

This research did not receive any specific grant from funding agencies in the public, commercial, or not-for-profit sectors.

### Data availability statement

Data included in article/supp. material/referenced in article.

### Declaration of interest’s statement

The authors declare no conflict of interest.

### Additional information

No additional information is available for this paper.
